# The efficacy and safety of fluconazole in preventing invasive fungal infection in very low birth weight infants: a systematic review and meta-analysis

**DOI:** 10.1186/s13052-023-01460-5

**Published:** 2023-04-27

**Authors:** Jinghong Xie, Jiayue Zeng, Shouyan Zheng

**Affiliations:** 1 Department of Pediatrics, Southwest Hospital of Army Medical University, Chongqing, China; 2 Department of Gastroenterology, the Affiliated Hospital of Southwest Medical University, Luzhou, Sichuan China

**Keywords:** Fluconazole, Fungal infection, Very low birth weight infants, Efficacy, Adverse reaction

## Abstract

This meta-analysis aimed to assess the efficacy and safety of fluconazole for the prevention of invasive fungal infections (IFI) in very low birth weight infants (VLBWI) and to provide a basis for the clinical use. A detailed search of Pubmed, Embase, Cochrane Library and other databases was performed to carefully screen eligible randomized controlled clinical studies to assess the safety and efficacy of fluconazole in very low birth weight infants in terms of the incidence of invasive fungal infections, fungal colonization rate, and mortality. Our research indicated that the application of fluconazole did not result in intolerable adverse reactions in patients. Fluconazole is effective in preventing invasive fungal infections in very low birth weight infants without serious adverse effects. The dose and frequency of fluconazole in very low birth weight infants still needs to be evaluated in consequent studies.

**Supplementary Information:**

The online version contains supplementary material available at 10.1186/s13052-023-01460-5.

## Introduction

Invasive fungal infection (IFI) refers to diseases in which fungi invade the human body, and even cause disseminated infections, leading to inflammatory reactions and tissue damage [1]. In recent years, with the increase of high-risk children in neonatal intensive care units (NICU), the widespread use of broad-spectrum antibiotics, the increase of various invasive procedures, and the application of advanced life support systems, IFI has become the main cause of infections in premature infants in NICU. At present, neonatal fungal infection is mainly caused by *Candida* [2]. *Candida* in normal people will not induce any discomfort symptoms, but *Candida* invasion seriously endangers the life safety of very low birth weight infants. The lighter the birth weight of newborns, the higher the incidence of IFI and the higher the mortality rate. In very low birth weight infants (VLBWI), the incidence of IFI is between 1% and 7.5%, and the mortality rate is as high as 19.3% [3]. The mortality rate of newborns with fungal infection is much higher than that of other newborns [4]. Among surviving children, about 60% are left with varying degrees of neurological sequelae. Clinical manifestations of neonatal fungal infection lack specificity, early diagnosis is difficult, and the mortality rate is high [5].

Therefore, in order to prevent the occurrence of invasive fungal infections in neonates, increasing research has been devoted to finding an effective treatment option. Fluconazole is widely used to treat various types of fungal infections, and several studies have compared the therapeutic effects of fluconazole in preventing invasive fungal infections caused by various *Candida* species (i.e., *Candida* albicans and other fungi), and the results have shown that it can reduce fungal colonization in different parts of the human body, such as the digestive tract, respiratory tract, and skin [4, 6]. These studies suggest that fluconazole may be an effective drug to control fungal infections in very low birth weight infants.

In recent years, some scholars have carried out systematic reviews on fluconazole in the prevention of IFI in very low birth weight infants [3]. However, most of these systematic reviews included small sample sizes, and were heterogeneous with few outcome measures, making it difficult to comprehensively illustrate the efficacy and safety of fluconazole in preventing IFI. In this study, we systematically searched the databases for clinical randomized controlled trials (RCT) on fluconazole in the prevention of invasive fungal infections in very low birth weight infants to comprehensively evaluate its efficacy and safety and provide a basis for subsequent related treatments.

## Materials and methods

### Strategy of literature search

According to the Preferred Reporting Items for Systematic Reviews and Meta-Analyses Guideline recommended by PRISMA, we examined all data from references using RCT for fluconazole prophylactic effect in VLBWI or preterm infants. We searched PubMed, MEDLINE, EMBASE, SCOPUS, Cochrane Library and other databases on October 2022. The primary search MeSH terms were as following: (((“Premature Infant” [Mesh]) OR “Very Low Birth Weight” [Mesh]) AND “fluconazole” [Mesh]) AND “fungal infection” [Mesh]. We also searched for some other related articles manually, ensuring thorough search.

### Inclusion and exclusion criteria

All articles included in this study should meet the following criteria: (i) Randomized controlled trials published in English; (ii) Study subjects were very low birth weight infant (< 1500 g); (iii) Intervention was oral/intravenous fluconazole prophylaxis; (iiii) Study presented primary outcomes and adverse events. Meanwhile, we excluded the following studies: (i) the types of study were medical record report, review or basic research; (ii) the study applied other antifungal agents instead of fluconazole; (iii) Study sample size less than 10 patients.

### Literature screening and data extraction

**Fig. 1:**
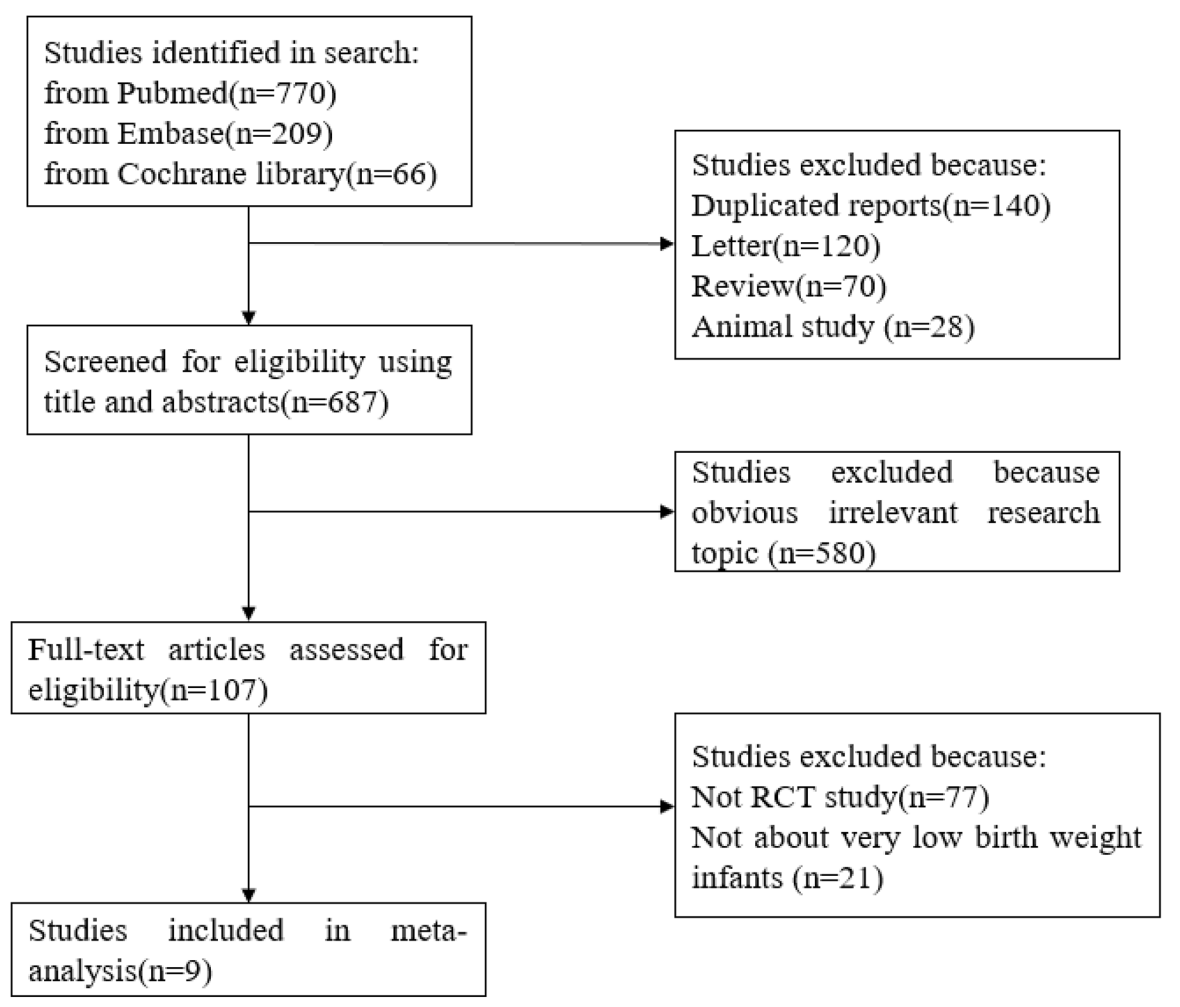
PRISMA flow chart of literature search process and study selection

Two investigators independently performed literature screening according to the screening criteria, first read the article title and abstract to exclude articles that clearly did not meet the inclusion criteria, and further read the full text. For articles with incomplete information, those who could not obtain complete data after contacting the authors were not included in this study. When the opinions of the two investigators were not uniform, a third investigator was invited to join the discussion and finally reach a consensus. Literature screening process and results are shown in Fig. 1. After completing the literature screening, the investigators performed data extraction on the articles that met the inclusion criteria, including the following items: author, publication year, study type, number of patients in the experimental and control groups, fluconazole dose and mode of administration, incidence of invasive fungal infections, and incidence of drug-resistant bacteria and other outcome measures.

### Quality Assessment

The quality of included studies was evaluated using the criteria provided by the Cochrane Handbook of Systematic Reviewers in terms of the following: randomization method, allocation scheme, blinding, reporting of loss to follow-up, selection bias, and other biases. If all criteria are low risk, its quality is the highest; if one or more types of risk are unknown, its quality is moderate; if one or more types of high risk, its quality is low.

### Statistical analysis

Meta-analysis was performed using Review Manager 5.3 software. *I*^2^ test was used for heterogeneity analysis among the included study results. When *I*^2^ ≤ 50% and *p* ≥ 0.1 indicated no significant heterogeneity among the studies, fixed effect model was used for analysis; when *I*^2^ > 50% and *p* < 0.1 indicated significant heterogeneity among the studies, the source of heterogeneity was further analyzed. After excluding the effect of significant clinical heterogeneity, random effect model was used for analysis. Relative risk (RR) was used as the effect index for enumeration data, standardized mean difference (SMD) was used as the effect index for measurement data, and *p* < 0.05 was considered statistically significant. Each effect size gave its point estimate and 95% confidence interval (CI). The significance level was set at *p* < 0.05.

## Results

### Study characteristics and quality

**Fig. 2:**
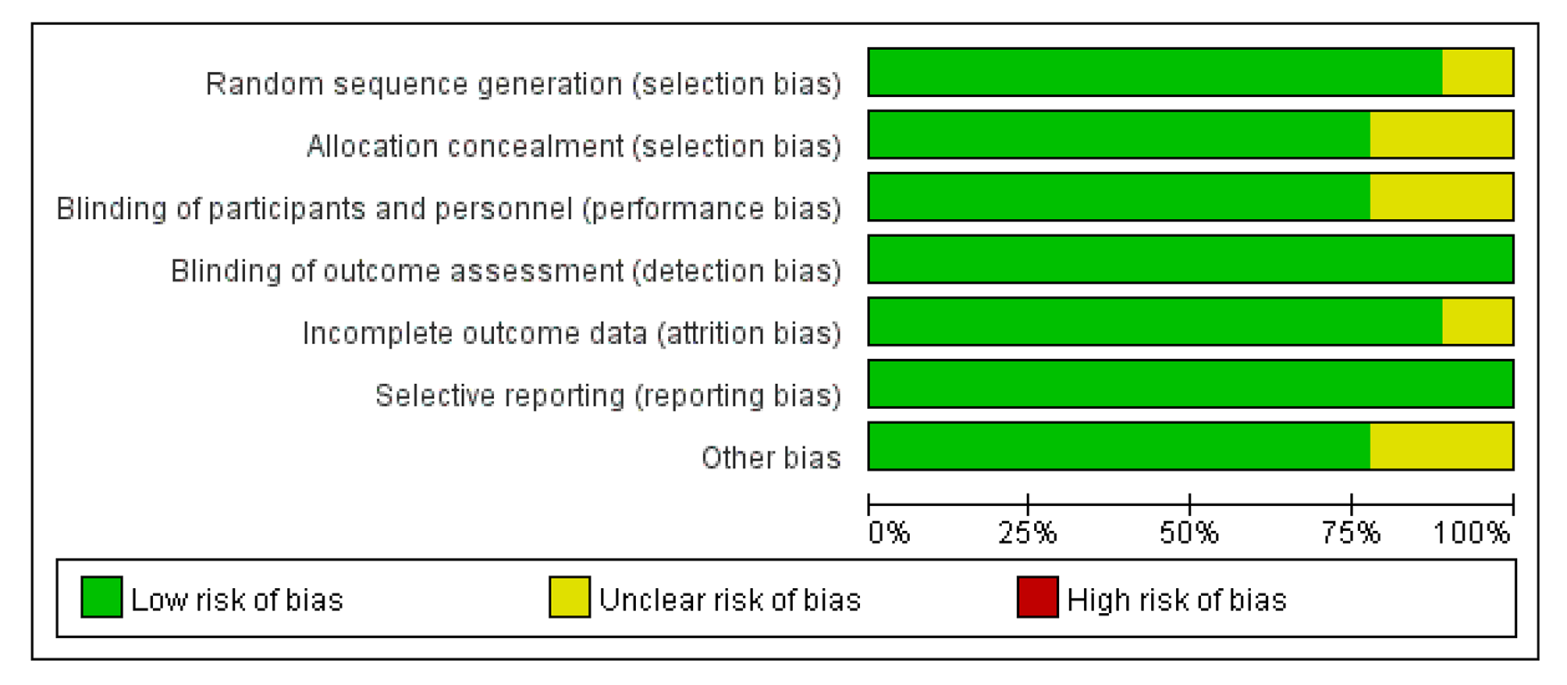
Risk of bias graph

**Fig. 3:**
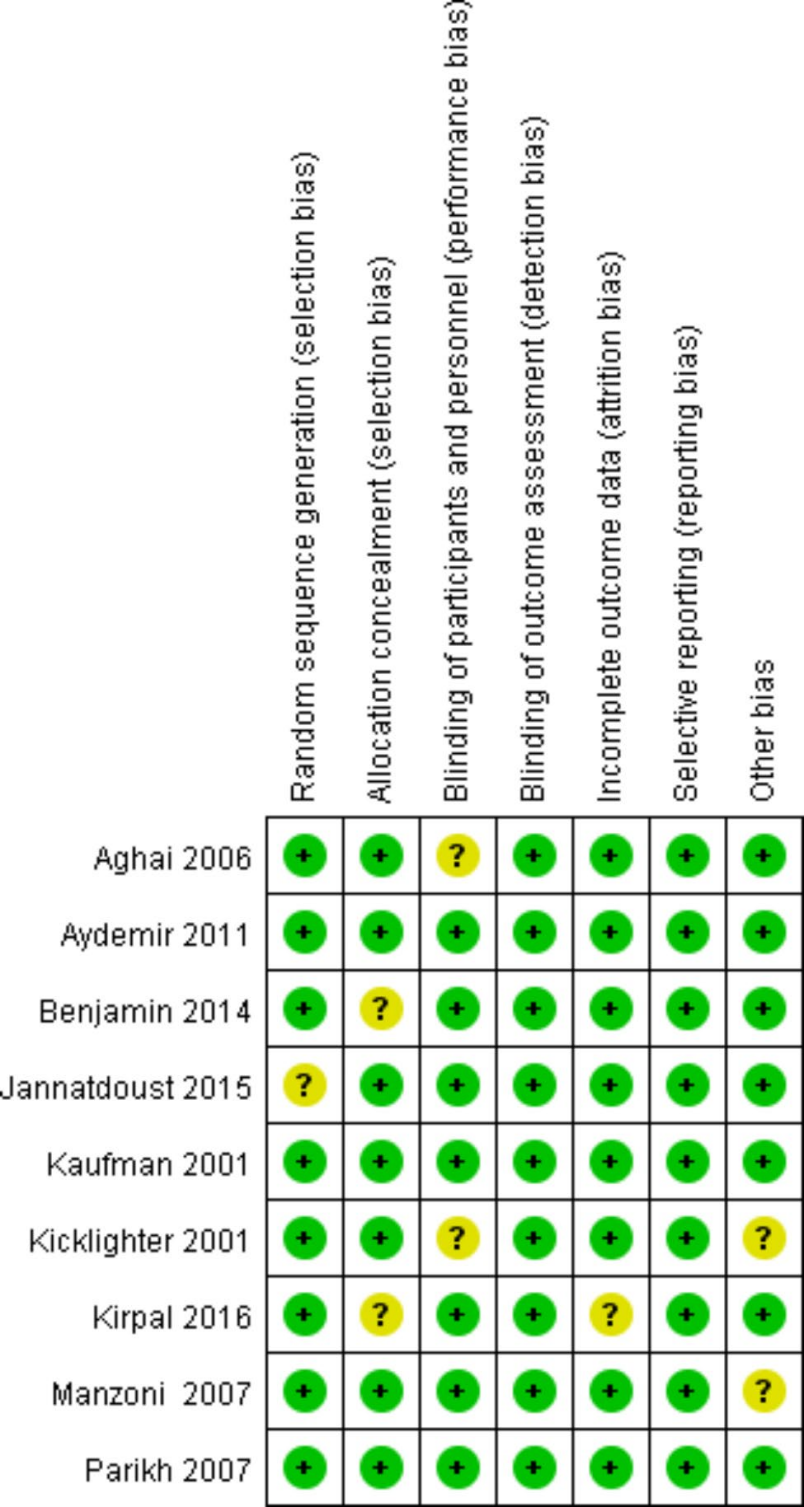
Risk of bias summary

According to the screening process shown in Fig. 1, a total of 1,045 articles were initially retrieved, and 9 articles were finally obtained after screening for inclusion in the study. A total of 1,635 VLBWI were included, 881 in the experimental group and 754 in the control group. Main characteristics of eligible studies are shown in Table 1. According to the criteria discussed previously, all the included trials were deemed to show a low risk of bias (Figs. 2 and 3).

**Table 1:** Characteristics of included studies

Authors	Year	Patients (control/ experimental)	Birth weight (control/ experimental, g)	Dose of fluconazole	Administration of therapy	Outcomes
Kicklighter et al. [7]	2001	50/53	919 ± 239/ 992 ± 258	6 mg/kg, every third day for 1 week then daily	Intravenous injection	a,b,c,
Kaufman et al. [8]	2001	50/50	744 ± 157/ 717 ± 150	3 mg/kg, every third day for two weeks; every 48 h for 2 weeks and then daily	Intravenous injection	a,b,c,
Parikh et al. [9]	2007	60/60	1280 ± 199/1210 ± 241	6 mg/kg, every 72 h till day 7 and subsequently every 24 h	Intravenous injection	a,b
Aydemir et al. [10]	2011	91/93	1102 ± 238/1127 ± 215	3 mg/kg, every third day	Intravenous injection	a,b,c,d
Manzoni et al. [11]	2007	106/216	1120 ± 270/1060 ± 245	112 patients received 6 mg/kg, 104 patients received 3 mg/kg, every third day for two weeks, then every 48 h	Intravenous injection	a,b,c,d
Aghai et al. [12]	2006	137/140	681 ± 169/749 ± 133	3 mg/kg, every 72 h for 2 weeks; every 48 h 2 weeks and daily 2 weeks	Intravenous injection	a,c
Benjamin et al. [13]	2014	173/188	640(573–700)/653(570–700)	6 mg/kg, twice weekly	Intravenous injection	a,c
Jannatdoust et al. [14]	2015	50/43	976 ± 203/969 ± 163	3 mg/kg, every 72 h till day 7 and subsequently every 24 h	Intravenous injection	c
Kirpal et al. [15]	2016	37/38	1220 ± 130/1250 ± 360	6 mg/kg, every 72 h till day 7 and subsequently every 24 h	Intravenous injection	a,c,d

a: Incidence of IFI; b: Fungal colonization rate; c: In-hospital mortality; d: Fungal infection-related mortality

### Incidence of IFI

**Fig. 4:**
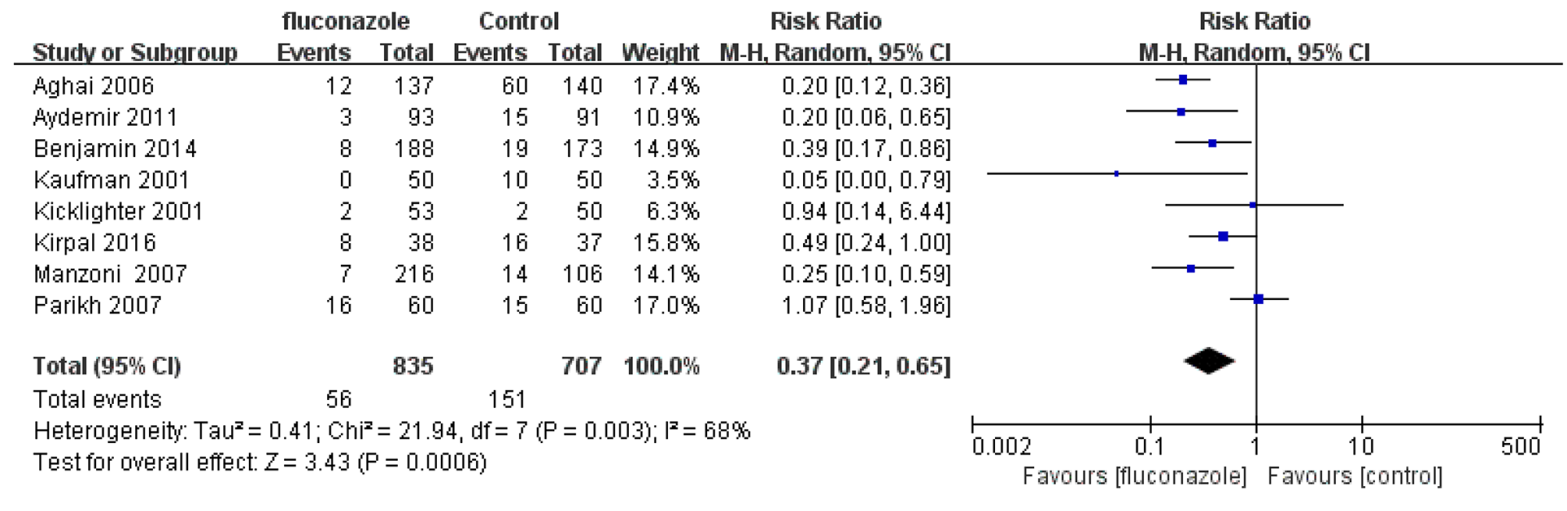
Forest plot based on incidence of IFI

A total of 8 studies observed the effect of prophylactic fluconazole on the incidence rate of IFI. The incidence rate of IFI in the experimental group and the control group was 6.7% (56/835) and 21.3% (151/707), respectively. The incidence rate of IFI in the experimental group was significantly lower than that in the control group (RR = 0.37; 95% CI: 0.21 ~ 0.65, P = 0.0006). The forest plot of meta-analysis is shown in Fig. 4.

### Fungal colonization rate

**Fig. 5:**
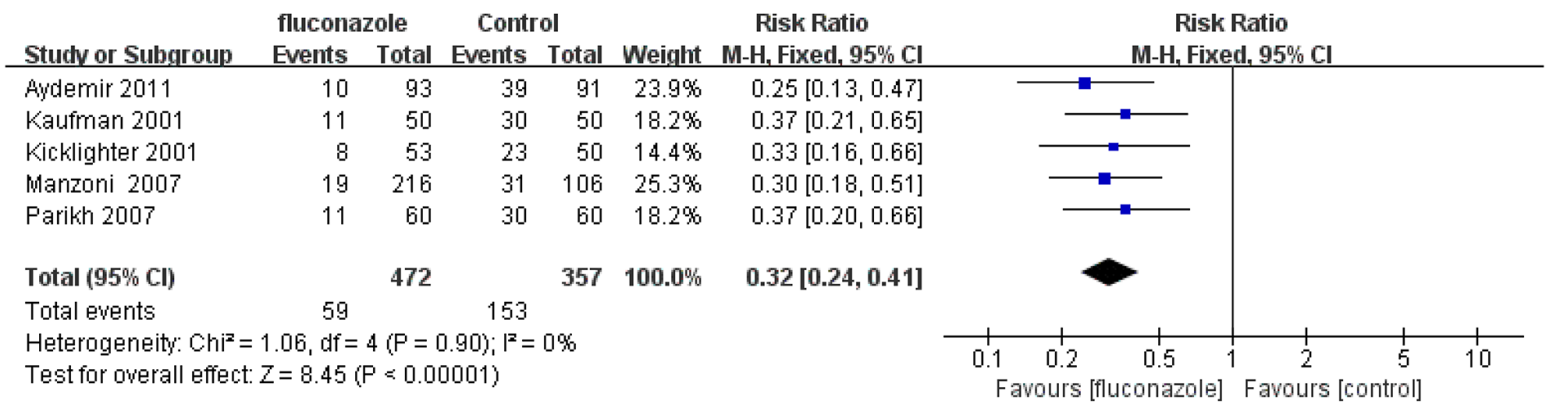
Forest plot based on fungal colonization rate

A total of 5 studies observed the effect of prophylactic fluconazole on the rate of fungal colonization, including colonization of the digestive tract, endotracheal tube or nasopharynx, and skin. The fungal colonization rate was 12.5% (59/472) in the experimental group and 42.9% (151/707) in the placebo group. The fungal colonization rate in the experimental group was significantly lower than that in the control group (RR = 0.32; 95% CI: 0.24 ~ 0.41, P < 0.00001). Forest plot is presented in Fig. 5.

### In-Hospital mortality

**Fig. 6:**
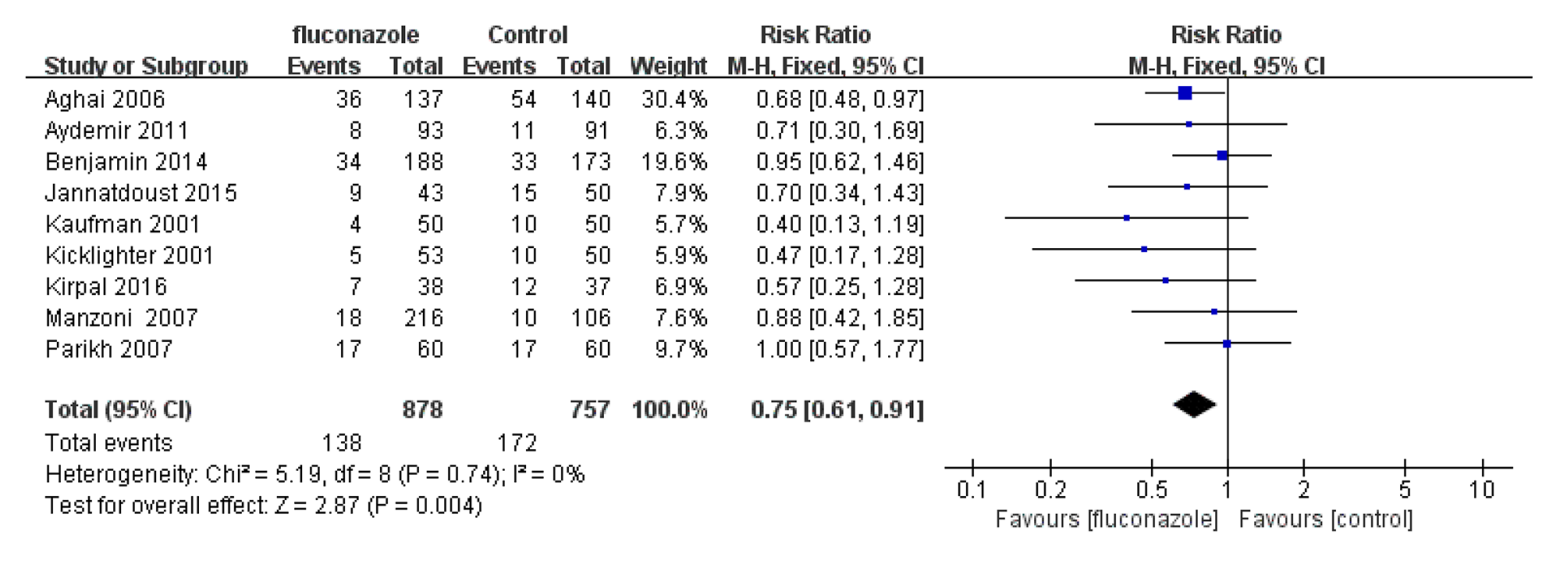
Forest plot based on in-hospital mortality

A total of 9 studies observed the mortality of hospitalized children. The in-hospital mortality of the experimental group and the placebo control group was 15.7% (138/878) and 22.7% (172/757), respectively. The in-hospital mortality of the experimental group was significantly lower than that of the control group (RR = 0.75; 95% CI: 0.61 ~ 0.91, P = 0.004). Forest plot is presented in Fig. 6.

### Fungal infection-related mortality

**Fig. 7:**
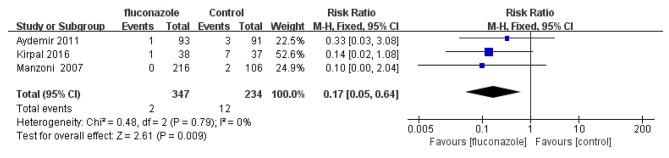
Forest plot based on fungal infection-related mortality

A total of 3 studies investigated the mortality related to fungal infection. The mortality related to fungal infection in the experimental group and placebo group was 0.6% (2/347) and 5.1% (12/234), respectively. The mortality related to fungal infection in the experimental group was significantly lower than that in the control group (RR = 0.17; 95% CI: 0.05–0.64, P = 0.009). The forest plot is shown in Fig. 7.

### Adverse reactions

Some complications occurred in both the experimental group and the control group, such as abnormal liver function, sepsis, necrotizing enterocolitis, bronchopulmonary dysplasia, patent ductus arteriosus and retinopathy of prematurity, but there were no significant differences in the incidence of complications between the two groups (P > 0.05). This suggests that fluconazole for antifungal therapy in very low birth weight infants does not bring additional side effects and does not cause significant physical harm to the child. Detailed statistics of adverse reactions are shown in Table 2.

**Table 2:** Adverse reactions of included studies

Adverse reactions	Control group (cases/toal paitents)	Experimental group (cases/toal paitents)	Heterogeneity test		RR(95%CI)	*p*
			*I* ^*2*^	*P*		
Abnormal liver function	66/608	64/739	0	0.82	0.90(0.67 ~ 1.20)	0.46
Sepsis	207/420	242/547	6%	0.36	0.94(0.82 ~ 1.07)	0.36
Necrotizing enterocolitis	43/420	46/547	0	0.60	0.90(0.60 ~ 1.34)	0.60
Bronchopulmonary dysplasia	135/370	164/497	55%	0.11	0.99(0.84 ~ 1.17)	0.93
Patent ductus arteriosus	61/329	83/454	4%	0.35	1.03(0.75 ~ 1.42)	0.86
Retinopathy of prematurity	72/420	84/547	28%	0.24	0.91(0.64 ~ 1.29)	0.60

## Discussion

In recent years, with the development of neonatal intensive care and the increase of critical neonatal treatment rate, the incidence of IFI in NICU is increasing day by day [16]. Candidemia is the most common fungal infection in NICU, can colonize, invade, and spread in the absence of any clinical manifestations, and often progresses to septic shock, meningitis, and even renal failure, increasing child mortality [17]. Because the clinical manifestations of IFI are atypical, difficult to diagnose, and easy to cause nervous system damage after the occurrence of fungal infection, timely treatment even could not reduce the incidence of nervous system damage, so the prevention of neonatal fungal infection is the key [18, 19]. In 1998, Kicklighter et al. [7] firstly used fluconazole prophylactically in VLBWI and found that it reduced the rate of fungal colonization without adverse reactions such as liver function impairment. Since then, many RCT studies have been conducted in NICU all over the world, but the conclusions are not completely consistent among studies, and their efficacy and safety are inconclusive [20].

In this study, we analyzed RCT studies using fluconazole to prevent invasive fungal infections in very low birth weight infants, and the results showed that fluconazole for the prevention of invasive fungal infections in very low birth weight infants significantly reduced the incidence of IFI and fungal colonization rate, which was similar to the findings of Austin et al. [21] In addition, the use of fluconazole also significantly reduced in-hospital mortality and fungal infection-related mortality in VLBWI, suggesting that the prophylactic use of fluconazole avoids the development of severe infections in VLBWI due to invasive fungal infections. Fluconazole is not used at the same dose and frequency in clinical practice, for example, 3 mg/kg and 6 mg/kg are currently used more frequently, and relevant studies have shown that both doses can significantly reduce the probability of IFI and mortality in VLBWI. There have also been reports on the use of fluconazole in dose studies, and Leonnart et al. [22] used three doses of 3 mg/kg, 4 mg/kg, and 6 mg/kg to prevent the occurrence of IFI, and their findings showed that there was no significant difference between the three doses in preventing the occurrence of IFI, and the probability of adverse reactions increased with higher doses.

At present, there is still a lack of conclusion on the safety of prophylactic fluconazole. The results of this meta-analysis showed that prophylactic fluconazole may lead to adverse reactions in some very low birth weight infants, such as Abnormal liver function, Sepsis, etc. Prophylactic fluconazole may increase drug-drug interactions, such as those with theophylline and thiazide diuretics, and increase the risk of theophylline toxicity and renal impairment, but there was no significant difference from the control group, and no children included in the study withdrew from the study because they could not tolerate adverse reactions, indicating that prophylactic fluconazole did not significantly increase the incidence of adverse reactions [23, 24]. Fluconazole is used in antifungal therapy and may increase fungal resistance to fluconazole, and Sarvikivi et al. [25] found that *Candida* albicans susceptibility to fluconazole was significantly reduced after up to 10 years of fluconazole use in the NICU. In the literature included in this study, resistance studies with fluconazole use have been reported in individual publications, and their results suggest that fluconazole doses routinely used in clinical practice do not enhance fungal resistance, which may be related to different doses and frequencies of use.

### Limitations

Limitations of our study are as follows: firstly, the included studies came from multiple sites, and the fungal infection rate and medication methods of the children in each study were different, which may have an impact on the results; secondly, some included subjects in the literature were ultra-low birth weight infants, which may have an impact on the study of the incidence of complications and mortality, because the possibility of complications was greater in children with lower body weight; in addition, the time span of the included studies was large, and there may be differences in the treatment and outcome evaluation methods during different time periods, which may lead to some heterogeneity.

## Conclusions

The results of this study showed that fluconazole had a positive effect in preventing invasive fungal infections in very low birth weight infants and significantly reduced the infection rate and mortality. Although the application of fluconazole may lead to drug-related adverse reactions, none of them were serious and tolerable, indicating that fluconazole treatment has a good safety profile. Further large multicenter randomized controlled studies may be conducted to assess the exact treatment modalities and dose compliance of fluconazole in very low birth weight infants.

## Electronic supplementary material

Below is the link to the electronic supplementary material.

Supplementary Material 1

Supplementary Material 2

## Data Availability

All data generated or analyzed during this study are included in this published manuscript.

## Abbreviations

IFI: Invasive fungal infections
VLBWI: Very low birth weight infants
NICU: Neonatal intensive care units
RCT: Randomized controlled trials
SMD: Standardized mean difference
RR: Relative risk
CI: Confidence interval.
