# Supplementary material for: The efficacy and safety of fluconazole in preventing invasive fungal infection in very low birth weight infants: a systematic review and meta-analysis
Source: Ital J Pediatr. 2023 Apr 27;49:51. doi: 10.1186/s13052-023-01460-5 (PMC10142176; doi:10.1186/s13052-023-01460-5)
Supplement: Supplementary file 2 — Supplementary Material 2 [file 13052_2023_1460_MOESM2_ESM.docx]

**D****etailed Responses to Reviewer**

**Response to reviewer #1:**

**Thank you very much for your valuable comments on our work. We have made several modifications accordingly in the revised manuscript.**

1. The review seems to be incomplete. The article "Fluconazole prophylaxis against fungal colonization and invasive fungal infection in very low birth weight infants. Parikh TB, Nanavati RN, Patankar CV, Rao S, Bisure K, Udani RH, Mehta P. Indian Pediatr. 2007 Nov;44(11):830-7" is an RCT that seems to fits the inclusion criteria chosen by the Authors. It's is not clear why it was not included. So that 120 patients are not included in the metanalysis.

#Answer: **Thank you for your comment. Actually, we included this article in the study as Ref.9.**

2. The dose of fluconazole and the tratment scheme in table 1 seems to be incomplete or wrong for 3 studies:

- Kicklight er et al (7): 6mg/kg, every third day for 1 week than daily

- Kaufman et al (8): 3mg/kg, every third day for two weeks; every 48h for 2 weeks and than daily

- Manzoni et al (11): 6mg/kg, every third day for two weeks; than every 48h*.*

#Answer: **Thank you very much for your valuable suggestions, which we have modified in the corresponding place in the text.**

3. References sometimes seem to be unsuitable for the text:

Some example:

- Line 191: the incidence of IFI in NICU is increasing day by day (16). The reference n. 16 is a case report (Raturi S, Chandran S, Viswanathan R. A case of Candida famata sepsis in a very low birth weight infant successfully treated with fluconazole following antifungal susceptibility testing. Medical Journal of Dr D Y Patil University. 2015; 8: 528)

- Line 200-201-202: Since then, many RCT 201 studies have been conducted in NICU all over the world, but the conclusions are not completely consistent among studies, and their efficacy and safety are inconclusive (20). The reference n. 20 is a case report (Yuan G, Tu Y, Liu L, Xu T. Successful fluconazole combined with caspofungin treatment of candida bloodstream infection in preterm infant: A case report. Medicine. 2021; 100: e28270)

#Answer: **Thank you for this recommendation regarding the references, which we have modified in the text.**

4. Table 1 reports that in Manzoni's RCT (11) patients were randomized between 6 mg/kg and placebo. Actually randomization was between 6 mg, 3 mg and placebo

#Answer: **sorry for this error and we' ve added the patients in the 3 mg group to the experimental group.**

5. In table 1 the numbers referring to patients and control are reversed (but data analysis is correct)

#Answer: **Sorry for this misunderstanding, we have adjusted the relevant content in Table 1.**

6. In table 1 patients and control of Manzoni's RCT are wrongly reported: 6 mg - 112 patients; 3 mg - 104 patients; placebo - 106 patients

#Answer: **Thank you for this comment, we have revised the corresponding parts in the text.**

**Response to reviewer #2:**

**Thank you for your support and encouragement of our research, which will prompt us to work harder and more diligently in our subsequent research.**

1. Would italicize genus/species (e.g. Candida, etc.) throughout.

#Answer: **Thank you for your advice and we have revised the content.**

2. Page 7, line 138 -would change "8 literatures" to "8 studies".

#Answer:  **We have amended the text in accordance with your suggestions.**

3. Page 8, line 159 - would change "9 literatures" to "9 studies".

#Answer: **We have revised the article in accordance with your suggestions.**

**We tried our best to improve the manuscript and made some changes in the manuscript.  These changes will not influence the content and framework of the paper. We appreciate for Editors/Reviewers’ warm work earnestly, and hope that the correction will meet with approval.
 Once again, thank you very much for your comments and suggestions.**

**With kindest regards,**

**Yours Sincerely,**

**Yunong Ao**
